# End stage renal disease‐induced hypercalcemia may promote aortic valve calcification via Annexin VI enrichment of valve interstitial cell derived‐matrix vesicles

**DOI:** 10.1002/jcp.25935

**Published:** 2017-05-24

**Authors:** Lin Cui, Nabil A. Rashdan, Dongxing Zhu, Elspeth M. Milne, Paul Ajuh, Gillian Milne, Miep H. Helfrich, Kelvin Lim, Sai Prasad, Daniel A. Lerman, Alex T. Vesey, Marc R. Dweck, William S. Jenkins, David E. Newby, Colin Farquharson, Vicky E. Macrae

**Affiliations:** ^1^ The Roslin Institute and Royal (Dick) School of Veterinary Studies University of Edinburgh Easter Bush Edinburgh United Kingdom; ^2^ Gemini Biosciences Ltd Liverpool Science Park Liverpool United Kingdom; ^3^ Institute of Medical Sciences University of Aberdeen Aberdeen United Kingdom; ^4^ Department of Cardiothoracic Surgery Royal Infirmary Hospital of Edinburgh (NHS Lothian) The University of Edinburgh Edinburgh United Kingdom; ^5^ University/BHF Center for Cardiovascular Sciences University of Edinburgh The Queen's Medical Research Institute Edinburgh United Kingdom

**Keywords:** Annexin VI, calcific aortic valve disease, calcification, matrix vesicles

## Abstract

Patients with end‐stage renal disease (ESRD) have elevated circulating calcium (Ca) and phosphate (Pi), and exhibit accelerated progression of calcific aortic valve disease (CAVD). We hypothesized that matrix vesicles (MVs) initiate the calcification process in CAVD. Ca induced rat valve interstitial cells (VICs) calcification at 4.5 mM (16.4‐fold; *p* < 0.05) whereas Pi treatment alone had no effect. Ca (2.7 mM) and Pi (2.5 mM) synergistically induced calcium deposition (10.8‐fold; *p* < 0.001) in VICs. Ca treatment increased the mRNA of the osteogenic markers *Msx2*, *Runx2*, and *Alpl* (*p* < 0.01). MVs were harvested by ultracentrifugation from VICs cultured with control or calcification media (containing 2.7 mM Ca and 2.5 mM Pi) for 16 hr. Proteomics analysis revealed the marked enrichment of exosomal proteins, including CD9, CD63, LAMP‐1, and LAMP‐2 and a concomitant up‐regulation of the Annexin family of calcium‐binding proteins. Of particular note Annexin VI was shown to be enriched in calcifying VIC‐derived MVs (51.9‐fold; *p* < 0.05). Through bioinformatic analysis using Ingenuity Pathway Analysis (IPA), the up‐regulation of canonical signaling pathways relevant to cardiovascular function were identified in calcifying VIC‐derived MVs, including aldosterone, Rho kinase, and metal binding. Further studies using human calcified valve tissue revealed the co‐localization of Annexin VI with areas of MVs in the extracellular matrix by transmission electron microscopy (TEM). Together these findings highlight a critical role for VIC‐derived MVs in CAVD. Furthermore, we identify calcium as a key driver of aortic valve calcification, which may directly underpin the increased susceptibility of ESRD patients to accelerated development of CAVD.

## 1 INTRODUCTION

Calcific aortic valve disease (CAVD), and subsequent aortic valve stenosis is the most common heart valve disease in the Western world (Newby, Cowell, & Boon, 2006; Nkomo et al., 2006). CAVD is currently considered an actively regulated and progressive disease, characterized by a cascade of cellular changes that initially cause fibrotic thickening, followed by an extensive calcification of the aortic valve leaflets. This in turn leads to significant aortic valve stenosis and eventual left ventricular outflow obstruction (Freeman & Otto, 2005), for which surgical replacement remains the only viable treatment option.

Patients with end‐stage renal disease (ESRD) have high circulating calcium (Ca) phosphate (Pi) levels, and develop extensive vascular calcification which directly contributes to cardiovascular morbidity (Jablonski & Chonchol, 2013; Moe & Chen, 2004; Zhu, Mackenzie, Farquharson, & Macrae, 2012). ESRD patients are highly susceptible to accelerated progression of CAVD (Perkovic et al., 2003; Rattazzi et al., 2013; Umana, Ahmed, & Alpert, 2003), however, this association has received little attention to date.

The pathophysiology of CAVD is complex, but shares similar mechanisms to physiological bone mineralization (Mohler et al., 2001). Valve interstitial cells (VICs), the most abundant cell type in the aortic heart valve, play a key role in CAVD progression. Numerous studies have demonstrated the ability of VICs to undergo osteogenic trans‐differentiation and calcification (Monzack & Masters, 2011; Osman, Yacoub, Latif, Amrani, & Chester, 2006). While the mechanisms underpinning this process have yet to be fully elucidated, it is highly plausible that parallels with chondrocyte and vascular calcification exist, whereby matrix vesicles (MVs) initiate the calcification process through interactions with collagen within the extracellular matrix (ECM) (Chen, O'Neill, Chen, & Moe, 2008; Kapustin & Shanahan, 2012; New et al., 2013).

MVs are membrane‐bound particles of cellular origin, ranging from 100 to 200 nm in diameter, and act as a nidus for hydroxyapatite nucleation (Cui, Houston, Farquharson, & MacRae, 2016). Vascular smooth muscle cell (VSMC)‐derived MVs have been associated with arterial calcification (Kapustin et al., 2011), with recent studies elucidating their composition, interrogating their function, and identifying these MVs as exosomes (Kapustin et al., 2011, 2015). However, the role of MVs in CAVD has yet to be fully determined.

This study aimed to characterize the role of MVs during aortic valve calcification. We have therefore, undertaken analysis of clinical CAVD tissues in conjunction with in vitro calcification studies in rat‐derived VICs to address the hypothesis that Ca and Pi induce aortic valve calcification through a MV‐mediated mechanism.

These findings yield novel insights into the mechanisms of CAVD and highlight a critical role for the involvement of VIC‐derived MVs. Furthermore, we identify Ca as a key driver of aortic valve calcification, which may directly underpin the increased susceptibility of ESRD patients to accelerated development of CAVD.

## 2 METHODS

### 2.1 Human tissues

Human aortic valve samples were obtained with appropriate ethical approval from patients undergoing valve replacement surgery (Ethics Number: 13/ES/0126). Research ethics committee approval (National Health Service West of Scotland Research Ethics Committee: 12/WS/0227) and the written and informed consent of all participants were obtained. The uncalcified control tricuspid valve tissue was from a 79‐year old female with aortic incompetence and generalized aortic dilatation with ascending aorta involvement. There was minor to moderate left ventricular impairment. Calcified tricuspid valve tissue was from a 67‐year old female with severe aortic stenosis detected by echocardiography. Angiography revealed severe distal left mainstem stenosis with further moderate disease in the left anterior descending artery and circumflex and a severe lesion within the diagonal artery. Additionally, there was a 90% stenosis in the mid right coronary artery. The aortic valves were removed at the time of aortic valve replacement with care taken to preserve the integrity of the valve architecture. Human tissue was used in this study in conformation with the declaration of Helsinki.

### 2.2 Primary rat VIC isolation

All animal experiments were approved by The Roslin Institute's Animal Users Committee and the animals were maintained in accordance with Home Office guidelines for the care and use of laboratory animals. Rats used were euthanized by cervical dislocation. Primary aortic VICs were isolated from aortic valve leaflets, dissected from the hearts of 5 weeks old male Sprague Dawley rats (Charles River Laboratories, Harlow, UK). Leaflets were initially digested in 425 U/ml collagenase type II (Worthington, Lakewood, NJ) for 10 min and washed in Hanks’ balanced salt five solution (HBSS; Life Technologies, Paisley, UK) to remove valve endothelial cells. The leaflets were subsequently digested with 425 U/ml collagenase type II for a further 2 hr. The cells subsequently obtained were re‐suspended in growth media consisting of Dulbecco's Modified Eagle Media (DMEM)‐Formula 12 (Life Technologies) supplemented with 10% foetal bovine serum (FBS; Life Technologies) and 1% gentamicin (Life Technologies) and cultured at 37 °C, in the presence of 5% CO_2_. Before experimentation, isolated VICs were expanded in growth media for 2–4 passages, and cells used for experiments were between passage 4–6.

### 2.3 Rat VIC line

The RVIC Sv40T (rat VIC‐derived cell line) was generated by transducing primary rat VICs with recombinant lentivirus expressing Simian virus (SV) 40 large T antigen. Cell immortalization is achieved by silencing the expression of the tumor suppressors such as p53 and retinoblastoma protein (Rb), through a siRNA expressed by the lentivirus (Capital Biosciences, Gaithersburg, MD).

### 2.4 Induction of calcification

Primary VICs were seeded in growth media at a density of 1.67 × 10^4^/cm^2^ in multi‐well plates. Calcification was induced as previously described in VSMCs (Reynolds et al., 2004). In brief, cells were grown to confluence (Day 0) before treatment with control (1.05 mM Ca/0.95 mM Pi) or test media; high calcium (Ca media; 2.7, 3.6, 4.5, or 5.4 mM Ca), high phosphate (Pi media; 2, 2.5, 3, 4, or 5.0 mM Pi), or both (CaPi media; 1.5–2.7 mM Ca/1.5–2.5 mM Pi). The standard CaPi media used was 2.7 mM Ca and 2.5 mM Pi. VICs were incubated for up to 5 days in 5% CO_2_ at 37 °C, and the medium was changed every 2nd/3rd day.

### 2.5 Determination of calcification

Calcium deposition was quantified based on a method previously described (Zhu et al., 2015; Zhu, Mackenzie, Millan, Farquharson, & Macrae, 2014). Briefly, cells were rinsed twice with phosphate buffered saline (PBS) and decalcified with 0.6 M HCl at room temperature for 2 hr. Free calcium was determined colorimetrically by a stable interaction with phenolsulphonethalein, using a commercially available kit (Randox Laboratories Ltd., County Antrim, UK), and corrected for total protein concentration (Bio‐Rad Laboratories Ltd, Hemel Hempstead, UK).

### 2.6 MV isolation

MVs were obtained by differential centrifugation using a modified MV isolation protocol (Reynolds et al., 2004). Primary VICs or RVIC Sv40T cells were cultured in control (serum free growth media) or in serum free standard CaPi media, for 16 hr at 37 °C. The media was subsequently aspirated and centrifuged at 3000 *g* for 20 min to pellet cell debris. The supernatant was then transferred to Beckman Coulter Ultra Clear™ ultracentrifuge tubes (VWR International Ltd, Lutterworth, UK) and centrifuged at 270,000 *g* for 1 hr at 4°C, using a Beckman Optima XL‐90 ultracentrifuge (Beckman Coulter, Buckinghamshire, UK). The supernatant was discarded and the pellets were re‐suspended in PBS. The concentration of pelleted MVs was measured using DC assay (Bio‐Rad).

### 2.7 Fluorescent immunocytochemical staining

Cell monolayers on glass coverslips, were fixed with 4% paraformaldehyde (PFA) and washed with PBS. Fixed cells were permeabilized with 0.3% Triton X–100 (Sigma, Dorset, UK) and incubated with anti‐α‐smooth muscle actin (α‐SMA; 1:100; Sigma), anti‐vimentin (1:900) or anti‐CD31 (1:900) (Abcam, Cambridge, UK) overnight at 4°C. After washing, cells were incubated with Alexa Fluor® 488 donkey‐anti‐rabbit antibody or Alexa Fluor® 594 goat‐anti‐mouse antibody (1:250; ThermoFisher Scientific, Northumberland, UK) for 1 hr in the dark. Glass coverslips were mounted onto slides with Prolong Gold Anti‐Fade Reagent contained DAPI (Life Technologies). Fluorescence signal was detected under a Leica DMRB fluorescence microscope (Leica Biosystems, Milton Keynes, UK). Control sections were incubated with non‐immune mouse or/and rabbit IgG (Sigma) (2 μg IgG/ml) in place of the primary antibody.

### 2.8 Transmission electron microscopy

Human valve tissue samples were cut into 1–2 mm^3^ pieces and fixed in 2.5% glutaraldehyde in 0.1 M sodium cacodylate buffer (pH 7.4). Samples were then processed with an automated routine tissue processor, Leica EMTP (Leica Biosystems) through a series of dehydration steps and penetration using TAAB 812 Epoxy resin (TAAB, Aldermaston, UK). The samples were then polymerized in TAAB embedding capsules (TAAB), sectioned at 0.5 μm, stained with toluidine blue, and scanned using a Zeiss Axioscan Z1 slide scanner (Carl Zeiss, Jena, Germany). Ultrathin sections (thickness 90 nm) were prepared on a Leica UC6 (Leica Biosystems) and contrasted with 5% uranyl acetate for 15 min, and lead citrate for 5 min, on a Leica AC20 (Leica Biosystems). For immunogold labeling, the samples were initially fixed in 0.5% glutaradehyde in 4% PFA. Following the same routine electron microscopy routine (using LR White Resin; TAAB), after ultrathin sectioning to 60 nm thick, the sections were collected on formvar‐carbon coated nickel grids, then heated in citrate buffer (pH 6) before letting the samples cool down. The slides were blocked with 5% bovine serum albumin (BSA; Sigma) for 1 hr before incubating with anti‐annexin VI antibody (1:1000; Santa Cruz Biotechnology, Dallas, TX), at 4 °C, overnight. On the following day, they were incubated with rabbit anti‐goat Gold antibody (1:300, Sigma) at room temperature, for 2 hr. Finally, they were contrasted with 5% uranyl acetate for 15 min, and lead citrate for 5 min as mentioned previously. Samples were viewed on a JEOL 1400/JEM plus (JEOL, Welwyn Garden City, UK) with AMT UltraVUE camera (AMT, Bury St. Edmunds, UK) and Gatan OneView camera (Gatan, Oxon, UK).

### 2.9 Histology and immunohistochemistry

Tissues were fixed in 10% neutral buffered formalin (NBF) for 24 hr before being dehydrated, embedded in paraffin wax, and sectioned (4 μm) using standard procedures as previously described (Zhu et al., 2016). Sections were de‐waxed in xylene and stained with Von Kossa or Alizarin red (Sigma) to visualize Ca deposition, and haematoxylin and eosin (H&E) to assess cell architecture. Immunohistochemistry was performed using the Vectastain ABC Kit (Goat IgG) (Vector Labs, Peterborough, UK) according to manufacturer's instructions. Sections were de‐waxed in xylene and de‐masked with citric acid based antigen unmasking solution (Vector Labs). Immunohistochemical analysis of CD68 (1:00 dilution, anti‐human mouse clone PG‐M1 m0876, DAKO, Glostrup, Denmark), was conducted after heat‐induced epitope retrieval using a citrate buffer (pH 6; Leica Biosystems) in a decloaking chamber. Sections were stained using a Leica Vision Biosystems Bond × immunostaining robot (Leica Biosystems). After blocking in peroxide for 10 min, sections were incubated with the specific primary anti‐human antibodies for 2 hr at room temperature. All incubation steps were followed by washing in Tris‐Buffered Saline and Tween 20 (TBS/T). CD68‐stained sections were then incubated for 15 min with prepolymer followed by 15 min with polymer conjugated horseradish peroxidase (HRP) for all antibodies, before 3,3′‐diaminobenzidine (DAB) visualization and H&E counterstain. Slides were mounted in Pertex (Cell Path, Powys, UK). For AnnexinVI staining, endogenous peroxidase, and non‐specific antibody binding were blocked before overnight incubation at 4 °C with 2 μg IgG/ml anti‐Annexin VI antibody (Santa Cruz Biotechnology). The sections were then washed in PBS, incubated with diluted biotinylated secondary antibody (1:200) for 30 min. After washing in PBS for 5 min, the sections were incubated for 30 min with Vectastain ABC Reagent (Vector Labs). The sections were then incubated with DAB substrate reagent (0.06% DAB, 0.1% hydrogen peroxide, in PBS) until the desired stain intensity developed. The sections were finally dehydrated, counterstained with H&E, and mounted in DePeX (Sigma). Control sections were incubated with goat IgG (2 μg IgG/ml) in place of the primary anti‐Annexin VI antibody.

### 2.10 Analysis of gene expression

RNA was extracted from VICs using RNeasy minikit (Qiagen, West Sussex, UK), according to the manufacturer's instructions. RNA was reverse transcribed and specific cDNAs were quantified by real‐time PCR using the SYBR green detection method as previously reported (Mackenzie et al., 2011; Staines, Zhu, Farquharson, & MacRae, 2014). Primers were obtained from Qiagen and Primer Design (Primer Design, Southampton, UK).

### 2.11 iTRAQ‐based quantitative proteomics analysis

VICs were incubated in control and standard CaPi media for 16 hr, and MVs were isolated by differential centrifugation from the supernatants to produce two biological replicates for both control and CaPi media. MVs (25 μg) were separated by one‐dimensional SDS–PAGE (4–12% Bis‐Tris Novex mini‐gel; Life Technologies), labeled using an iTRAQ Reagents Multiplex kit–four–plex (AB Sciex UK Ltd, Warrington, UK) and subjected to LC MS/MS analysis (LTQ‐Orbitrap Velos; ThermoFisher Scientific) in two technical replicates following an established protocol previously described (Broek Vander, Chalmers, Stevens, & Stevens, 2015).

### 2.12 iTRAQ data analysis

The raw mass spectrometric data files obtained were collated into a single data set using Proteome Discover version 2.0 (ThermoFisher Scientific) and the Mascot search engine version 2.4 (www.matrixscience.com). Proteins were selected if they had at least two unique peptides and a MASCOT peptide score ≥21 (which corresponds to the threshold value for a 95% confidence level). Physical and functional interaction properties of the identified proteins were predicted with Ingenuity Pathway Analysis (IPA; Ingenuity System, Redwood City, CA). Computational prediction of a vector of a signaling pathway (up or down) was based on mapping of particular positive or negative regulators associated with the pathway. Data presented show the differential expression of calcifying MVs relative to the control MVs, where *q‐*values represent adjusted *p*‐values derived from the false discovery rate.

### 2.13 Western analysis

Cultured cells were lysed in RIPA buffer (ThermoFisher Scientific). Immunoblotting was undertaken as previously described (Zhu, Mackenzie, Millan, Farquharson, & MacRae, 2013). PVDF membranes (Sigma) were probed overnight at 4 °C with anti‐Annexin VI antibody (1:000, Santa Cruz Biotechnology), anti‐PARK7 antibody (1:2000, Abcam), or anti‐RHO A/C (1:2000, Abcam) in 5% BSA. The membranes were then washed in TBS/T and incubated with anti‐goat IgG‐peroxidase (Dako) for 1 hr (1:3000 in 5% BSA). The immune complexes were visualized by enhanced chemiluminescence (ECL; GE Healthcare, Buckinghamshire, UK). Semi‐quantitative assessment of band intensity was achieved using ImageJ image analysis software (National Institutes of Health, Bethesda, MA).

### 2.14 Statistical analysis

Two‐sample Student's *t*‐test was used to analyze the significance between two sets of data. For more than two groups, one‐way analysis of variance (ANOVA) using the general linear model (GLM) incorporating pairwise comparisons was performed. Data are presented as mean ± standard error of the mean (S.E.M). All statistical analysis was performed using Minitab 17 (Minitab Inc., Coventry, UK). *p *< 0.05 was considered to be significant, and *p‐*values are represented as: *p < 0.05; **p < 0.01; ***p < 0.001.

## 3 RESULTS

### 3.1 MVs are present in calcified human aortic valve tissue

**Figure 1:**
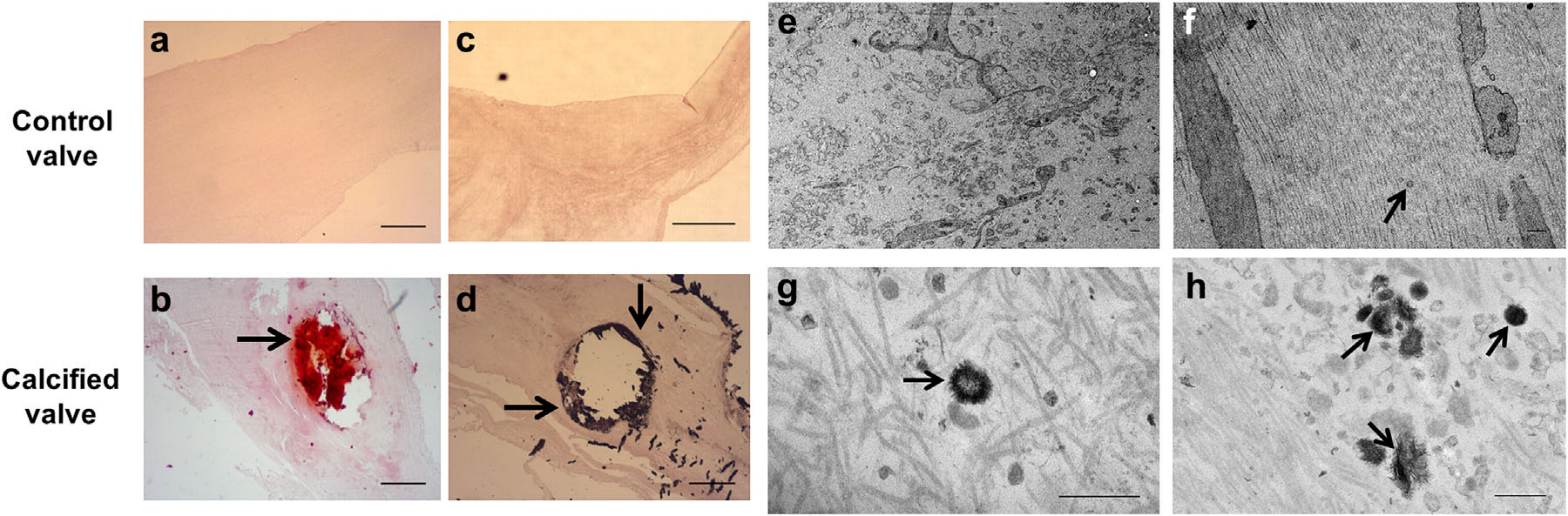
MV deposition in the ECM of human calcified aortic valve tissue (a,b). Aortic valve calcification was confirmed by Alizarin red and Von Kossa staining (c,d). Arrows indicate positive areas of staining. Transmission electron microscopy shows (g,h) MVs with spindle‐like projections resembling hydroxyapatite crystal needles in calcified tissue, and (e,f) “empty” vesicle structures in control tissue (arrows). Scale bars = 500 μm (Alizarin red/Von Kossa); 500 nm (TEM)

**Figure 2:**
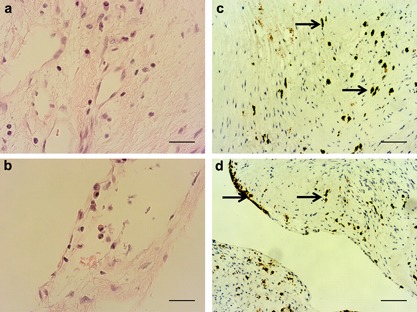
Inflammation in CAVD tissues. (a,b) H&E staining of stenotic aortic valve tissues showing disorganized ECM, and (c,d) CAVD tissues stained for CD68, a monocyte and macrophage marker showing the presence of inflammation by positive macrophage staining (arrows). Scale bars = 500 μm (H&E); 200 μm (CD68)

To ascertain whether MVs are central to the etiology of CAVD, structural studies were initially undertaken to determine the presence of MVs in clinical tissues. Calcification of aortic valve tissue was confirmed by Alizarin red and Von Kossa staining (Figure 1a–d). Transmission electron microscopy showed accumulation of vesicular bodies, ranging from 150 to 250 nm in diameter in the ECM of CAVD tissue. Many vesicles displayed spindle‐like projections of hydroxyapatite, as previously reported in MVs derived from chondrocytes (Figure 1g,h; [Garimella, Sipe, & Anderson, 2004]). Vesicles displaying this distinctive morphology were not observed in the control tissue, despite the presence of “empty” vesicles of comparable sizes (Figure 1e,f). Assessment of tissue morphology using H&E staining revealed disorganized ECM in CAVD tissue (Figure 2a,b). Further staining using CD68 antibodies revealed the presence of inflammation in CAVD samples (Figure 2c,d).

### 3.2 Ca regulates VIC calcification

**Figure 3:**
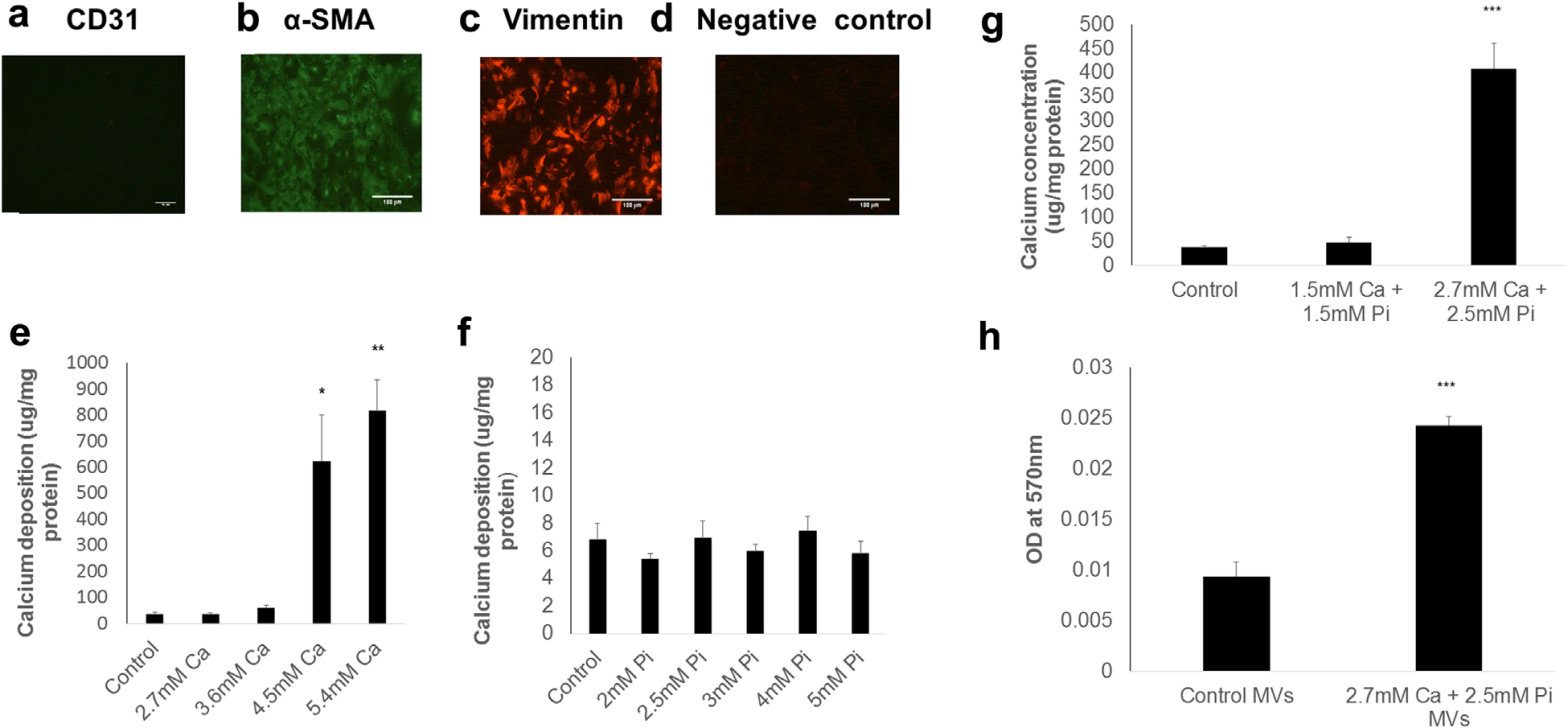
Calcium regulates VIC calcification. Immunofluorescence staining of rat primary VICs demonstrates negative staining for (a) the endothelial cell marker CD31 and positive staining for (b) smooth muscle actin (SMA; green) and (c) vimentin (red). (d) Representative image of negative control (NC) staining. Calcium deposition in VICs treated with (e) calcium (Ca) alone (2.7–5.4 mM) (f) phosphate (Pi) alone (2–5.0 mM) and (g) calcium and phosphate in combination (1.5–2.7 mM Ca/1.5–2.5 mM Pi). (h) Calcium content of MVs derived from VICs cultured in control and standard CaPi medium. Results are presented as mean ± S.E.M. *p < 0.5; **p < 0.01; ***p < 0.001 compared with control; *n* = 6

Initial studies confirmed that rat primary VICs isolated in the present investigation were free from endothelial contamination. Cells were negative for the endothelial cell marker, CD31 (Figure 3a). In addition, cells showed positive staining for both α‐SMA (green; Figure 3b) and vimentin (red; Figure 3c), in agreement with previous reports (Latif et al., 2015; Liu, Joag, & Gotlieb, 2007).

In ESRD, systemic Ca and Pi concentrations typically exceed 2.4 mM and 2.0 mM, respectively (Reynolds et al., 2004). To understand the accelerated progression of CAVD in patients with ESRD, it is essential to appreciate the calcification potential of VICs exposed to comparable concentrations of these established drivers of ECM calcification. Ca potently induced VIC calcification at 4.5 mM (16.4‐fold; *p* < 0.05; Figure 3e) whereas Pi treatment alone had no effect (Figure 3f). Notably, the treatment of VICs with Ca and Pi together had a synergistic effect on VIC calcification (2.7 mM Ca and 2.5 mM Pi; 10.8‐fold; *p* < 0.001; Figure 3g). The standard CaPi media induced a significant up‐regulation of Ca content in VIC‐derived MVs compared to control media (2.6‐fold; *p* < 0.001; Figure 3h).

**Figure 4:**
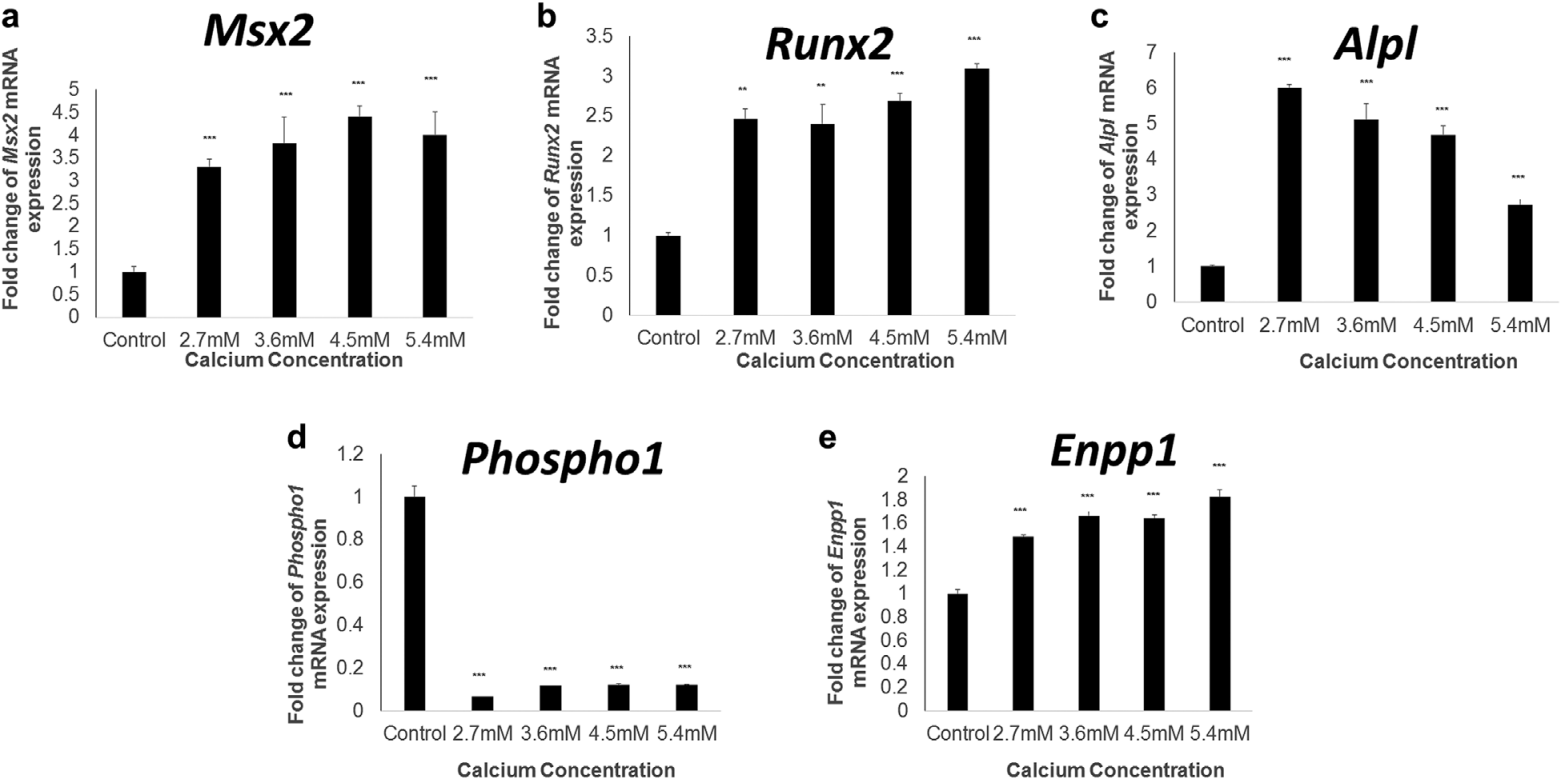
Calcium regulates osteogenic gene marker expression in VICs. Fold change in the mRNA expression of (a) *Msx2* (b) *Runx2* (c) *Alpl* (d) *Phospho1* and (e) *Enpp1* in VICs treated with calcium (1.5–5.4 mM) for 5 days. Results are presented as mean ± S.E.M. **p < 0.01; ***p < 0.001 compared to control; *n* = 6

A minimum concentration of 2.7 mM Ca treatment induced a significant increase in the mRNA expression of the osteogenic markers *Runx2*, *Msx2*, and *Alpl* (*p* < 0.01; Figure 4a–c). Intriguingly, a counter‐intuitive reduction in the mRNA expression of the MV enriched phosphatase, *Phospho1* (*p *< 0.001; Figure 4d), with a concomitant increase in the mineralization inhibitor *Enpp1* (*p* < 0.001; Figure 4e).

### 3.3 Proteomics analysis of MVs from calcified VICs reveals calcification regulators and exosome markers

To elucidate the specific structural and functional features of MVs derived from calcified VICs, we analyzed their protein composition using iTRAQ‐based quantitative mass spectrometry analysis (Supplementary Table). Remarkably, a number of established exosomal proteins were enriched in MVs isolated from VICS cultured with standard CaPi calcification media, including CD9, CD63, LAMP‐1, and LAMP‐2 (Table 1). Of further interest was the concomitant up‐regulation of the calcium‐binding Annexins (I, II, III, IV, V, VI, VII, and XI) (Table 2), which have been previously shown to specifically accumulate in chondrocyte and VSMC‐derived mineralization competent MVs in a calcium‐dependent manner (Kapustin et al., 2011; Wang, Xu, & Kirsch, 2003).

**Table 1:** Up‐regulation of exosomal proteins in calcified VIC‐derived MVs

Protein Name	Gene name	UniProt ID	Mean ratio	*Q*‐value
CD 9 antigen	CD9	B1WBM0	8.99	0
CD 63 antigen	CD63	F1LPA7	3.78	0.0035
Lysosomal associated membrane protein 1	LAMP‐1	P14562	49.60	0
Lysosomal associated membrane protein 2	LAMP‐2	F1LLX8	41.81	0
Tumor susceptibility gene 101	Tsg101	F1LRB7	11.45	0
Heat shock 70 kDa protein 8	Hspa8	P63018	12.50	0
Annexin V	Anxa5	Q66HH8	31.17	0
Heat shock protein 90, class A member 1	Hsp90aa1	P82995	13.81	0
Enolase 1	Eno1	Q5BJ93	20.17	0
Tyrosine 3‐monooxygenase/tryptophan 5‐monooxygenase activation protein, zeta polypeptide	Ywhaz	A0A0G2JV65	6.35	0

**Table 2:** Up‐regulation of Annexins in calcified VIC‐derived MVs

Protein	Gene name	UniProt ID	Mean ratio	*Q*‐value
Annexin I	Anxa1	P07150	52.28	0
Annexin II	Anxa2	Q07936	43.54	0
Annexin VII	Anxa7	Q6IRJ7	34.46	0
Annexin VI	Anxa6	Q6IMZ3	31.39	0
Annexin V	Anxa5	Q66HH8	31.17	0
Annexin XI	Anxa11	Q5XI77	24.67	0
Annexin IV	Anxa4	Q5U362	20.31	0
Annexin III	Anxa3	F1M0L7	9.69	0.018

### 3.4 Co‐localization of Annexin VI with MVs in human CAVD tissue

**Figure 5:**
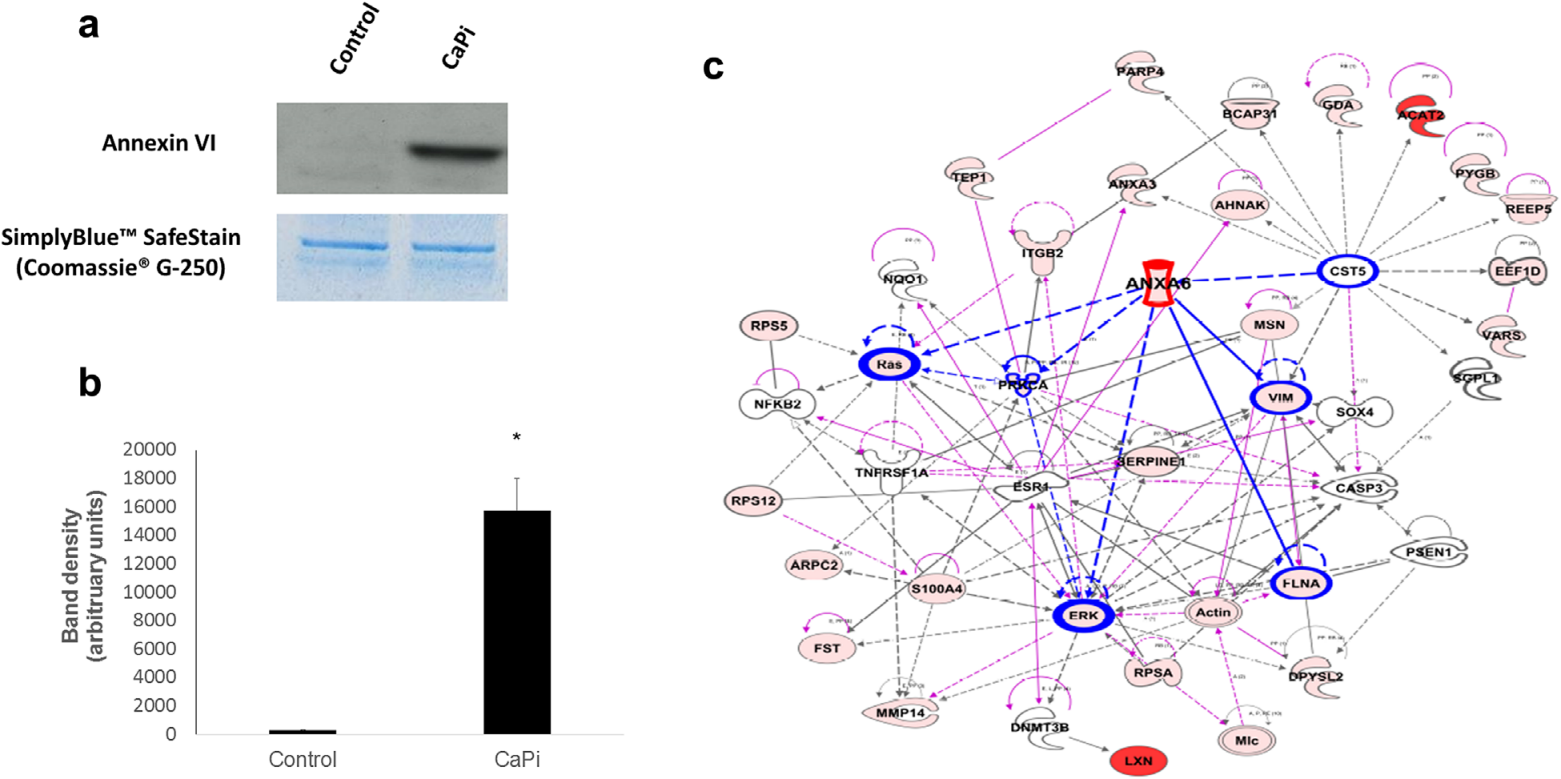
Enriched Annexin VI expression in calcifying VIC‐derived MVs and calcified aortic valve tissue. (a) Representative image of western blotting for Annexin VI in MVs isolated from VICs cultured in control and standard CaPi medium and (b) densitometry quantification showed increased expression of Annexin VI in calcifying VIC‐derived MVs. Results are presented as mean ± S.E.M. *p < 0.05 compared to control; *n* = 3. (c) Ingenuity pathway analysis showing the associations between Annexin VI and other proteins within the MV. The functions mapped by the dataset are represented by pink shadowed blocks. Functional interconnections between the proteins are shown by pink arrows and blue lines. The blue lines represent direct associations with Annexin VI. Dashed lines represent predicted associations

**Figure 6:**
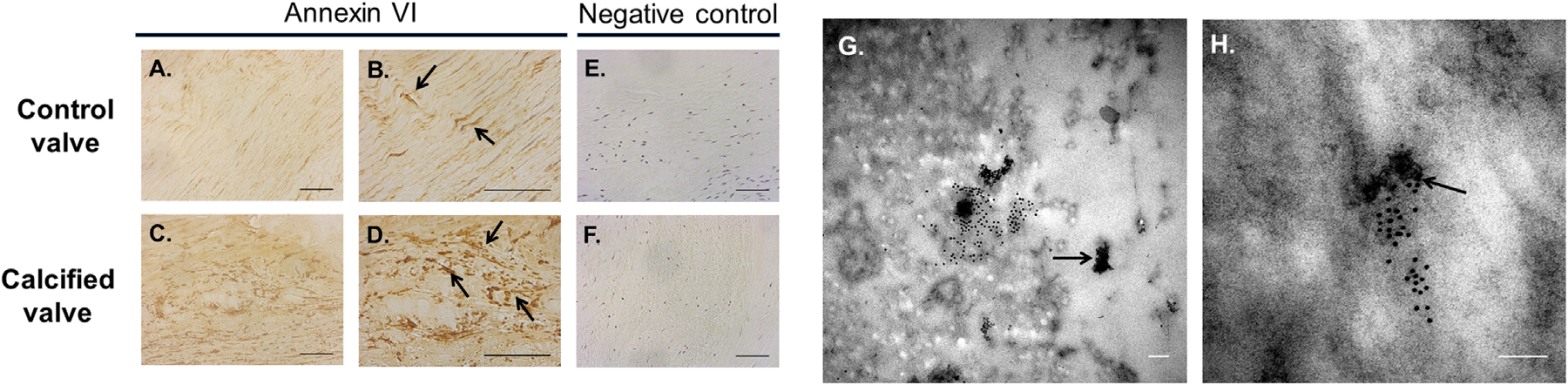
Co‐localization of Annexin VI with MVs in human CAVD tissue. Increased Annexin VI expression was observed in (c,d; arrows) calcified aortic valve compared to (a,b) control tissue. (e,f) Representative images of negative control staining. (g,h) Immunogold labeling showed co‐localization of Annexin VI with MVs (arrows) in calcified aortic valve tissue. Scale bar = 50 μm (Immunohistochemistry); 100 nm (TEM)

Following recent reports establishing that Annexin VI is required for MV mediated VSMC calcification (Kapustin et al., 2011), we further investigated the role of this calcium‐binding protein in VIC calcification. Immunoblotting studies confirmed that treatment with standard CaPi calcification media induced a significant up‐regulation of Annexin VI expression in rat VIC‐derived MVs compared to control media (Figure 5a,b). IPA revealed novel putative interactions between Annexin VI and other proteins within the MV, including vimentin and filamin A (FLNA) (Figure 5c). The enrichment of Annexin VI in calcifying MVs was further supported by immunohistochemical assessment of calcified human aortic valves, which revealed higher expression of Annexin VI (Figure 6c,d), compared to control valve issue (Figure 6a,b). Subsequent immunogold labeling confirmed the co‐localization of Annexin VI expression with MVs in CAVD tissue (Figure 6g,h).

### 3.5 Identification of novel putative MV pathways

**Figure 7:**
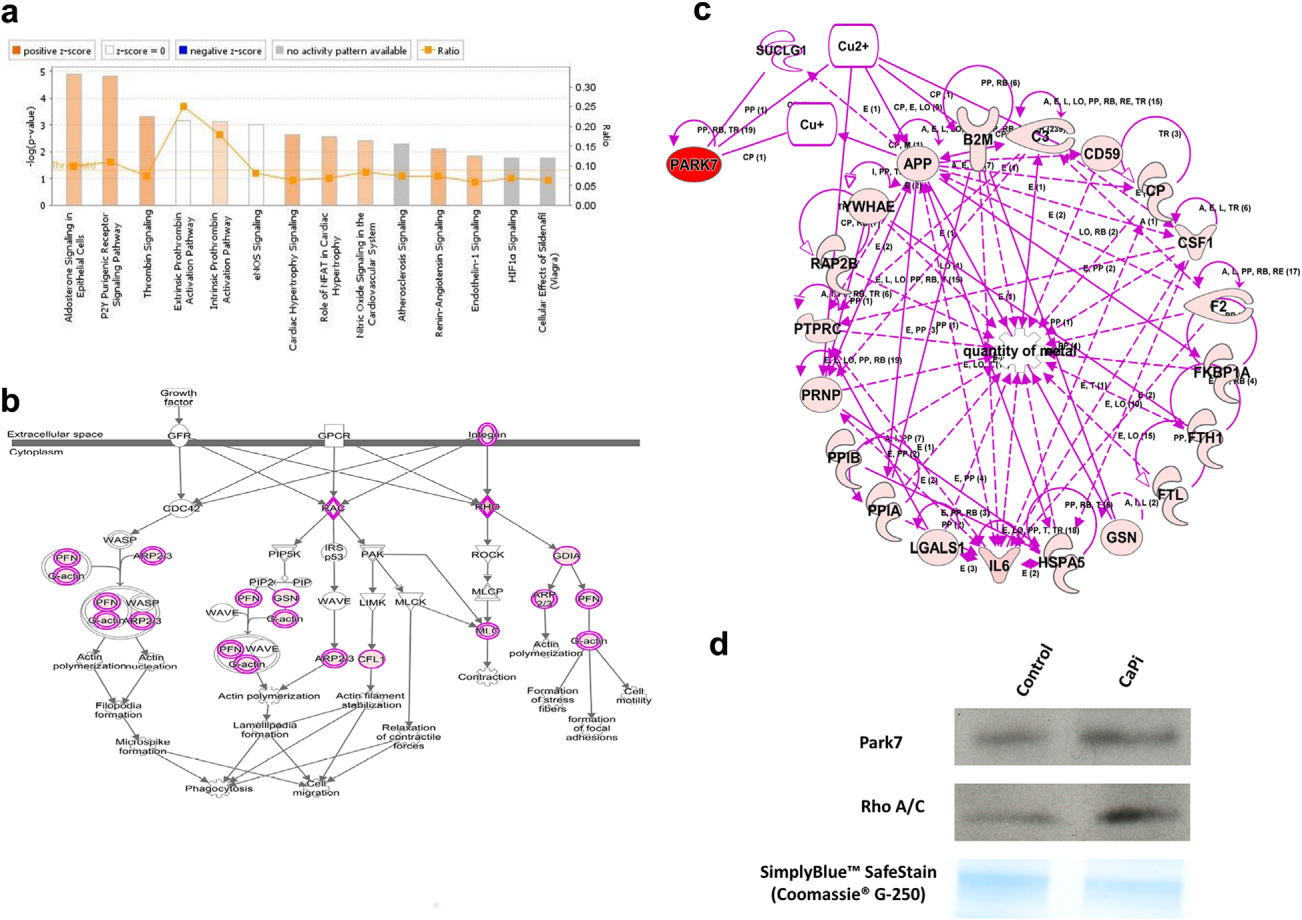
Ingenuity pathway analysis reveals the up‐regulation of canonical signaling pathways relevant to cardiovascular function. (a) Canonical pathways relevant to cardiovascular function associated with proteins that are differentially expressed in calcifying VIC‐derived MVs. Yellow data points indicate the ratio of the identified differentially expressed proteins and known protein pathways. The ratio value reflects the proportion of functions mapped by the dataset to total number of functions in a pathway. (b) Canonical pathway for Rho signaling. The functions mapped by the dataset are represented by pink shadowed blocks. (c) Pathway analysis showing the associations between PARK7 and metal binding. The functions mapped by the dataset are represented by pink shadowed blocks. Functional interconnections between the proteins are shown by pink arrows. Dashed lines represent predicted associations. (d) Western blot analysis for Park7 and Rho A/C in MVs isolated from RVIC Sv40T‐cell line cultured in control and standard CaPi medium

With a view to highlighting novel biological processes underpinning MV‐mediated calcification in VICs, we used IPA to identify canonical pathways, biological functions, and networks of interacting proteins. Key pathways crucial to cardiovascular function were identified, including aldosterone, P2Y purinergic receptor signaling and thrombin signaling pathways (Figure 7a), Rho signaling (Figure 7b) and metal binding (Figure 7c). Of particular interest was the discovery of several copper‐associated proteins within the calcifying MVs (Figure 7c), including Protein deglycase DJ‐1 (PARK7) which functions as a redox‐sensitive chaperone and as a sensor for oxidative stress (Shendelman, Jonason, Martinat, Leete, & Abeliovich, 2004; Zhou, Zhu, Wilson, Petsko, & Fink, 2006). Further studies conducted using western blot analysis on RVIC Sv40T‐derived MVs confirmed the higher expression of Rho A/C and Park7 in calcifying MVs (Figure 7d).

## 4 DISCUSSION

Given the significant contribution of increased circulating Ca to the arterial medial calcification associated with ESRD, in conjunction with reports showing the concentration of Ca in calcified aortic valves to exceed 13.5 mM/g tissue (Dahm et al., 2000), a more complete understanding of the role of calcium in aortic valve calcification is essential.

We report for the first time that VIC calcification in vitro can be driven by elevated Ca levels, with a concomitant increase in the expression of osteogenic markers *Msx2*, *Runx2*, and *Alpl*. Pi treatment alone was surprisingly ineffective at calcification induction. Intriguingly, a notable synergistic effect of Ca and Pi in combination on VIC calcification was observed, corroborating earlier reports demonstrating that elevated Ca induced human VSMC calcification in vitro with a synergistic effect of Ca and Pi (Reynolds et al., 2004). Abnormalities in Ca and Pi metabolism may therefore, directly underpin the increased susceptibility of ESRD patients to accelerated progression of CAVD.

This study offers new insight into the role of MVs in cardiovascular disease and provides direct evidence to suggest that MVs contribute to the pathological process of aortic valve calcification. Our experiments have established that MVs are released by viable VICs, particularly in the presence of elevated levels of extracellular Ca and Pi. These data support recent mechanistic studies of VSMC‐derived MVs (Kapustin et al., 2011), which proposed that Ca‐loaded vesicles are released from cells to protect against the cytotoxic effects of intracellular Ca overload (Fleckenstein‐Grün, Thimm, Czirfuzs, Matyas, & Frey, 1994; Hsu & Camacho, 1999). Furthermore, our ultrastructural analyses have identified the presence of MVs in calcified human aortic valves. These data extend previous reports demonstrating the existence of MVs in medial arterial calcification (Kim, 1976), and atherosclerotic intimal plaques (New et al., 2013).

Recent data have challenged current views on the plasma membrane origin of MVs, identifying VSMC‐derived MVs as exosomes emanating from intracellular MV bodies (Kapustin et al., 2015). In the present study, iTRAQ‐based quantitative mass spectrometry analysis revealed that a number of established exosomal proteins were enriched in MVs isolated from calcified VICs. It is therefore, conceivable that unlike bone‐derived MVs, which are understood to be released through polarized budding (Cui et al., 2016), MVs implicated in cardiovascular calcification are of exosomal origin. Further studies are therefore, required to more fully characterize the regulation of MV biogenesis in aortic valve calcification.

MVs produced by calcifying chondrocytes and VSMCs have been shown to contain Annexin II, V, and VI, the membrane‐associated proteins known to mediate Ca influx into MVs (Balcerzak et al., 2008; Kapustin et al., 2011; Xiao et al., 2007). Here we demonstrate the up‐regulation of Annexins I, II, III, IV, V, VI, VII, and XI in calcifying VIC‐derived MVs, underscoring the significance of the Annexin family in the process of cardiovascular calcification. We reveal for the first time not only the presence of Annexin VI expression in calcified human aortic valve tissue, but also the co‐localization of Annexin VI expression within calcifying MVs. These data suggest a novel role for Annexin VI in the regulation of calcium homeostasis and MV release in CAVD, building on previous reports demonstrating a specificity of Annexin V for macrophage‐derived MVs (New et al., 2013) and Annexin VI for VSMC‐derived MVs (Chen et al., 2008; Kapustin et al., 2011) in aortic calcification. Indeed, the cell type‐dependent enrichment of annexins in calcifying MVs may be a key factor in the regulation of MV release and calcification potential within the cardiovascular system.

Despite recent advances in our knowledge, the full mechanisms underpinning CAVD have yet to be fully elucidated. This study has clearly demonstrated differential expression of novel proteins and pathways in calcifying VIC‐derived MVs, many of which were not reported in previous proteomic analyses of calcifying MVs (Balcerzak et al., 2008; Kapustin et al., 2011; Xiao et al., 2007). Numerous proteins associated with aldosterone and thrombin canonical pathways were identified in our analysis, which have both been previously linked to accelerated vascular calcification (Borissoff et al., 2012; de Rita, Hackam, & Spence, 2012; Hillaert et al., 2013). P2Y purinergic receptor signaling was also up‐regulated, which has been recently linked to aortic medial calcification in chronic kidney disease (CKD) patients through the activation by uridine adenosine tetraphosphate (Up4A) (Schuchardt et al., 2012). Additionally, we observed increased expression of several members of the Rho signaling pathway, which has been previously shown to be associated with VIC calcification in vitro (Gu & Masters, 2011). We also identified novel pathways associated with PARK7, which is involved in copper‐dependent signaling and functions as a redox‐sensitive chaperone and as a sensor for oxidative stress (Shendelman et al., 2004; Zhou et al., 2006). Furthermore, PARK7 is involved in activating androgen receptor‐dependent transcription (Kolisek et al., 2015), and may therefore, play a key role in the acceleration of vascular calcification by testosterone as recently reported by our laboratory (Zhu et al., 2016).

Together these findings yield novel insights into the mechanisms of aortic valve calcification and highlight a critical role for VIC‐derived MVs in CAVD. Furthermore, we identify calcium as a key driver of aortic valve calcification, which may have important implications for ESRD patients. This data may provide a stepping stone toward understanding the mechanisms of early calcification in renal failure patients as well as the establishment of new targets for the development of future therapeutic strategies for CAVD.

## DISCLOSURE

Authors wish to declare that there are no conflicts of interest.

## Supporting information

Additional Supporting Information may be found online in the supporting information tab for this article.

Supporting Table S1.
